# Machine learning assisted prediction of the Young’s modulus of compositionally complex alloys

**DOI:** 10.1038/s41598-021-96507-0

**Published:** 2021-08-25

**Authors:** Hrishabh Khakurel, M. F. N. Taufique, Ankit Roy, Ganesh Balasubramanian, Gaoyuan Ouyang, Jun Cui, Duane D. Johnson, Ram Devanathan

**Affiliations:** 1 Department of Mathematics, The University of Texas at Arlington, Arlington, TX 76019 USA; 2 Pacific Northwest National Laboratory, Richland, WA 99354 USA; 3 Department of Mechanical Engineering and Mechanics, Lehigh University, Bethlehem, PA18015 USA; 4 Ames Laboratory, United States Department of Energy, Ames, IA 50011 USA; 5 Department of Materials Science and Engineering, Iowa State University, Ames, IA 50011 USA

**Keywords:** Structural materials, Theory and computation

## Abstract

We identify compositionally complex alloys (CCAs) that offer exceptional mechanical properties for elevated temperature applications by employing machine learning (ML) in conjunction with rapid synthesis and testing of alloys for validation to accelerate alloy design. The advantages of this approach are scalability, rapidity, and reasonably accurate predictions. ML tools were implemented to predict Young’s modulus of refractory-based CCAs by employing different ML models. Our results, in conjunction with experimental validation, suggest that average valence electron concentration, the difference in atomic radius, a geometrical parameter λ and melting temperature of the alloys are the key features that determine the Young’s modulus of CCAs and refractory-based CCAs. The Gradient Boosting model provided the best predictive capabilities (mean absolute error of 6.15 GPa) among the models studied. Our approach integrates high-quality validation data from experiments, literature data for training machine-learning models, and feature selection based on physical insights. It opens a new avenue to optimize the desired materials property for different engineering applications.

## Introduction

The conventional alloying method almost always starts with one or two principal metallic elements and advances by incorporation of different alloying elements to engineer desired mechanical and chemical properties^1–3^. Therefore, the mechanical and chemical properties of the synthesized alloy remain controlled by the principal elements. For instance, Fe is the principal element in steels, Cu/Zn in brass, Ni/Co in superalloys and Ti in titanium alloys^4–6^. About 15 years ago, Yeh and Cantor^7,8^ introduced a novel alloy concept known as high entropy alloys (HEA) that consist of multiple-principal elements (N = 5 or more elements) in near equiatomic percentages. The increased complexity introduces higher configurational entropy (growing as k_B_T NlnN, where T is the temperature) compared to conventional alloys. As the number of elements N increases, the number of pairs grows as ~ N^2^ and raises the probability of favorable pair-driven formation enthalpy, which introduces a complex-chemistry effect (often referred to as a “cocktail effect”). The mixing of multi-principal elements generally introduces four core effects, such as, high mixing entropy, lattice distortions, slow diffusion, and a “cocktail” effect, which result in a simple microstructure and excellent mechanical properties^9–13^. Further study revealed that several HEAs, such as the Mo_0.5_AlNbTa_0.5_TiZr system, did not overcome the enthalpic contributions due to comparatively lower configurational entropies and featured the formation of secondary phases instead of just solid solution phases. Therefore, a more preferred terminology for such alloy systems has emerged, with the more general naming and definition called CCAs^14,15^ which is the naming convention used throughout this paper.

The number of elemental compositions is much higher in CCAs than that of traditional metallic alloys because CCAs comprise multiple-principal elements^16^. Moreover, a broader range of compositional space provides an opportunity to improve mechanical properties, such as Young’s modulus, yield strength, and hardness. However, it is extremely challenging to select the appropriate composition by trial-and-error experiment or intuition^17^. Atomistic modeling, such as molecular dynamics (MD), density functional theory (DFT), and thermodynamic modeling have been devoted to study phase stabilization, solidification, and crystallization kinetics of CCAs^18–25^. These techniques are computationally expensive, challenging to apply to the study of large polycrystalline samples, time consuming, and hence cannot be used on a large scale to narrow down the search space. Moreover, the variety of microstructures gives rise to complex and computationally expensive calculations compared to traditional alloys and hence it is challenging to predict the chemistries and compositions for a target property.

Nowadays, data-driven research and more specifically ML, which is widely used in self driving cars^26^, image classification^27^, web-searches^28^, and fraud detection^29^, is also employed to solve different challenges in materials science^30^. For instance, Zhang et al.^19^ found that atomic size difference (δ), mixing entropy ($\Delta S_{mix}$) and enthalpy ($\Delta H_{mix}$) are the most important features in phase selection of HEAs. Singh et al.^31–33^ used high-throughput DFT to predict properties through the chemical ranges and revealed correlations with valence electron concentration (VEC), size-difference (bandwidth) and vacancies. Roy et al.^34^ proposed that the average melting temperature (*T*_*m*_) is the most important feature to predict the Young’s modulus of low, medium and high entropy alloys. Recent efforts utilizing ML^35^ considered two additional features such as, Pauling electronegativity difference and difference in VEC and used a neural network (NN) to predict the phases that form in these CCAs. Thus, different features control each property of the alloy and the importance of features varies from property to property.

Here, we have employed different tree-based ensemble ML models, linear regression ML models, kernel-based ML models to predict the Young’s modulus of CCAs consisting of refractory elements. This work initially identified VEC, average melting temperature and difference in atomic radii as the most important physical properties that control the Young’s modulus of CCAs. The study compared the relative merits of different ML models for a training set of refractory alloy data that was gathered from published literature. The model prediction was then validated against the Young’s modulus measured for 32 new alloys synthesized and tested as part of this work. The findings offer considerable promise for alloy down selection based on ML models validated against high-quality experimental data of known provenance.

## Methodology

### Training data collection and feature selection

Data on Young's modulus for CCAs were collected from existing literature^34,36–38^. Two different data sets were used for model training. The first data set contains 154 alloys with a mixture of refractory and non-refractory alloys. The second data set contains 96 refractory alloys of Mo, Nb, Ta, W, mixed with some other elements like Al, Cr and Ni. Both datasets are presented in Tables 1 and 2 in the supplementary section. The goal of using two different data sets (one with a mixture of refractory and non-refractory alloys and the other with only refractory alloys) was to examine the effect of the elemental composition of training data on the reliability of the prediction with respect to experimentally synthesized validation data.

For the features that were used to train the ML models, we calculated 11 feature values of these alloys. These features are listed in Table 1. Past studies have shown that all of these features have a direct effect on the Young’s modulus for any alloy. To obtain these features, we collected data on features identified from domain knowledge, such as Pauling electronegativity, VEC, lattice constant, melting temperature, mixing enthalpy and atomic radii. Then we used Python language scripts to calculate the features mentioned in Table 1.

**Table 1:** Features of alloys considered in this analysis.

Feature	Description	References
$\Delta \chi = \sqrt {\sum\nolimits_{i = 1}^{n} {C_{i} (x_{i} - \overline{x})^{2} } }$	Difference in Pauling electronegativity $\chi_{i}$ weighted by composition *Ci* for each element *i*	^39^
$\Delta H_{mix} = \sum\nolimits_{i = 1,i \ne j}^{n} {4H_{ij} C_{i} C_{j} }$	Mixing Enthalpy derived from enthalpies *H*_*ij*_ for a pair of elements i and j	^40^
$\Delta S_{mix} = - R\sum\nolimits_{i = 1}^{n} {(C_{i} \ln C_{i} )}$	Mixing entropy; *R* is the universal gas constant	^41^
$\delta = \sqrt {\sum\nolimits_{i = 1}^{n} {C_{i} \left( {1 - \frac{{r_{i} }}{{\overline{r}}}} \right)}^2 }$	Difference in atomic radius *r*_*i*_ weighted by composition *Ci* for each element *i*	^42^
$\Delta a = \sqrt {\sum\nolimits_{i = 1}^{n} {C_{i} (a_{i} - \overline{a})^{2} } }$	Difference in lattice constants $a_{i}$ weighted by composition *Ci* for each element *i*	Analogues to $\delta$
$\Delta T_{m} = \sqrt {\sum\nolimits_{i = 1}^{n} {C_{i} (T_{i} - \overline{T})^{2} } }$	Difference in melting temperatures $T_{i}$ weighted by composition *Ci* for each element *i*	Analogues to $\Delta \chi$
$\lambda = \frac{{\Delta S_{mix} }}{{\delta^{2} }}$	A geometrical parameter	^43^
$\Omega = \frac{{T_{m} \Delta S_{mix} }}{{\left| {\Delta H_{mix} } \right|}}$	Parameter for predicting solid state formation	^42^
$T_{m} = \sum\nolimits_{i = 1}^{n} {C_{i} Ti}$	Average melting temp calculated by rule of mixture	^44^
$a_{m} = \sum\nolimits_{i = 1}^{n} {C_{i} a_{i} }$	Average lattice constant calculated by rule of mixture	^44^
$VEC = \sum\nolimits_{i = 1}^{n} {C_{i} (VEC)_{i} }$	Average valence electron concentration calculated by rule of mixture	^40^

**Figure 1:**
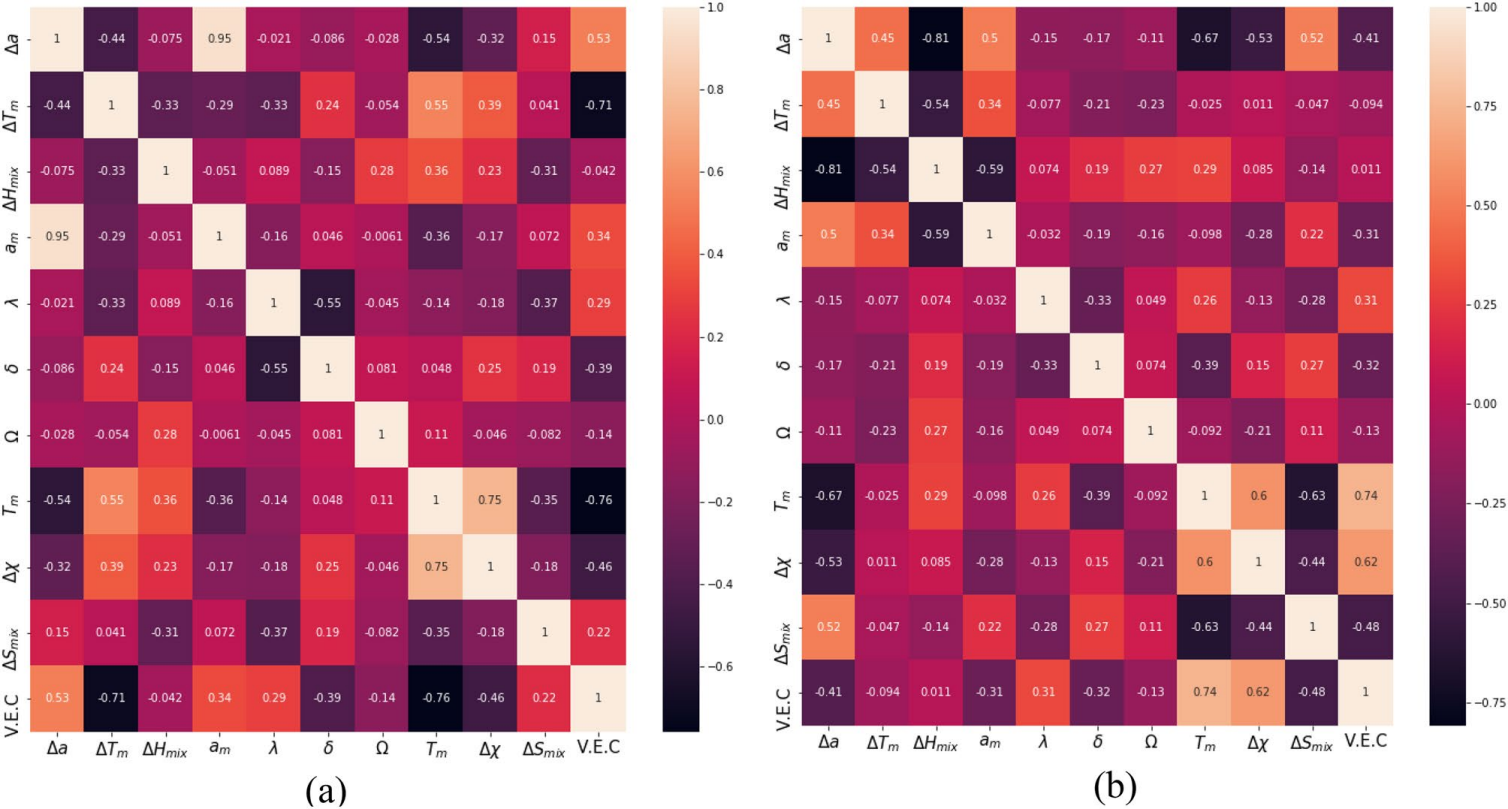
(a) PCC for data with both refractory and non-refractory alloys, (b) PCC for data with only refractory alloys. A value close to 1 or − 1 indicates positive or negative correlation, respectively.

To see the association between the features, we examined the Pearson correlation coefficients (PCC). Figure 1 shows the PCC for the mixed alloys data set and for the refractory alloys data set. In the PCC “heatmap”, *P* = + 1 indicates a strong positive correlation and *P* = − 1 indicates a strong negative correlation. Figure 1 indicates the absence of any significant correlation amongst any pair of features except $\Delta$a and a_m_ from Fig. 1a. However, the ML models we considered here can deal with the multicollinearity, and hence this correlation will not have any significant impact on the predictions. Therefore we considered all the features in the model.

### Validation data preparation and Young’s modulus measurement

An experimental data set was used to validate the final model predictions. The validation set consisted of 32 alloys in the Mo-based family of refractory CCAs, including Mo, Ta W, Ti, Zr, Al, Cr. The validation alloys used in the study were prepared at Ames Lab Materials Preparation Center in the form of thin metal plates/foils. The alloys (1.5 g each) with selected compositions were synthesized by arc melting using a 32-cavity arc melting system (MTI corp, SP-MAM32). The actual compositions of the alloys after arc melting were quantified by energy dispersive spectroscopy (EDS). The densities of the samples were measured by Archimedes measurement. The arc-melted buttons were then sliced by electrical-discharge machining into near-cylinder shapes (two parallel sides) with thicknesses of ~ 3 mm. The elastic modulus values were measured on the cylinders by the ultrasonic pulse-echo technique using a digital ultrasonic thickness gauge (Olympus, 38DL PLUS).

### Machine learning models construction

To predict the Young's modulus, four tree based ensemble methods i.e. Gradient Boosting, Ada Boost, Extreme Gradient Boost (or XGBoost), Random Forest (RF), two linear models i.e. LASSO regression, Ridge regression, two kernel based methods i.e. Gaussian Process Regression and Support Vector Machine (SVM) models were used. These models were trained for the two sets of data separately. Once the data was collected and the feature values were selected for both data sets, the 8 ML models were trained on both the data sets. We obtained 16 models, 8 for the larger data set with both the refractory and non-refractory alloys and 8 for the smaller data set with only refractory alloys. Five-fold cross-validation was used to determine the errors. The cross-validation approach is better than the train-test split approach as it gives more robust estimation of the errors. There exist many good metrics to quantify the predictive strength of the model like root-mean-squared (RMS) error, mean-squared error, mean-absolute error (MAE), and the coefficient of determination R^2^. We chose to use the MAE as our metric as it most closely represents the format of error as reported in most experimental measurements. Additionally, we also reported the R^2^ values for the optimized models.

The errors were minimized by performing hyper-parameter optimization using the grid-search algorithm. This algorithm works by determining the test error for all possible combinations of the supplied hyper-parameter values. Out of all combinations, the one with the least error was selected for our model. Each of the algorithms has a different set of hyper-parameters. Once the best hyper-parameters were selected, the optimized model using those hyperparameters was used to make predictions for our validation set whose Young’s modulus had been experimentally measured. Finally, the uncertainty of the predictions i.e. standard deviations was calculated by Bootstrapping method by resampling 100 times for each case. All of the above-mentioned tasks like cross-validation and grid search were performed using the scikit-learn^45^ library in Python. For our study, we employed all the ML models through the scikit-learn machine learning library for the Python language^46^. The XGBoost model was implemented through the library created by Tianqi Chen^47^.

## Results and discussion

### Model optimization

The ML models were first trained on both data sets. The hyper-parameters were optimized and then the training and validation error were calculated using five-fold cross-validation. We used these hyper-parameters to construct our final optimized models. The optimized hyperparameters are presented in the supplementary section (Table 3 in the supplementary section). These hyperparameters were used to predict the Young’s modulus for the unseen data i.e. the experimentally synthesized validation data set. The cross-validated MAE and R^2^ values for all the models are presented in Table 2. From Table 2 it is clear that the performance of the Gradient Boosting model is superior to other models both in terms of accuracy (i.e., the MAE is lower and R^2^ is higher than any other models) and robustness (i.e., the standard deviation of cross-validation is lower). Because of this excellent performance, we will discuss the feature importance and prediction of Young’s modulus generated by the Gradient Boosting model.

**Table 2:** Optimized hyperparameter and cross-validated MAE and R^2^ for both data sets.

Model	Cross-validated training MAE (GPa)		Cross-validated test MAE (GPa)		Cross-validated training R^2^		Cross-validated test R^2^	
	Refractory and non-refractory dataset	Refractory dataset	Refractory and non-refractory dataset	Refractory dataset	Refractory and non-refractory dataset	Refractory dataset	Refractory and non-refractory dataset	Refractory dataset
Gradient Boosting	0.42 ± 0.26	0.36 ± 0.16	10.37 ± 1.59	6.15 ± 1.19	0.99 ± 0.003	0.99 ± 0.007	0.71 ± 0.080	0.90 ± 0.036
XGBoost	0.33 ± 0.28	1.04 ± 0.48	10.32 ± 1.50	6.68 ± 1.22	0.99 ± 0.003	0.99 ± 0.008	0.70 ± 0.076	0.89 ± 0.038
RF	5.63 ± 0.59	5.54 ± 0.63	13.53 ± 1.50	9.00 ± 1.08	0.95 ± 0.009	0.96 ± 0.010	0.68 ± 0.076	0.89 ± 0.031
Ada Boost	12.79 ± 0.94	5.54 ± 0.84	18.02 ± 1.57	9.31 ± 1.53	0.86 ± 0.021	0.97 ± 0.011	0.62 ± 0.080	0.88 ± 0.051
SVM	14.78 ± 1.61	1.90 ± 0.57	17.83 ± 1.99	6.41 ± 1.39	0.64 ± 0.060	0.97 ± 0.013	0.54 ± 0.074	0.87 ± 0.053
Lasso regression	19.29 ± 1.44	17.53 ± 1.14	21.09 ± 1.64	18.16 ± 1.41	0.60 ± 0.060	0.72 ± 0.049	0.51 ± 0.076	0.67 ± 0.172
Ridge regression	19.37 ± 1.40	33.18 ± 3.32	21.24 ± 1.95	33.34 ± 3.26	0.60 ± 0.057	0.075 ± 0.007	0.51 ± 0.082	0.018 ± 0.065
Gaussian process	33.52 ± 1.90	34.08 ± 3.28	33.81 ± 1.92	34.55 ± 3.32	4.95 E−6 ± 4.9 E−7	1.35 E−5 ± 2.2 E−6	0.04 ± 0.028	0.090 ± 0.067

In our data sets, tree-based ensemble type models perform better than other models to predict Young’s modulus. Ensemble type algorithm showed better performance in other studies to predict materials properties^34,48,49^. Ensemble methods are meta algorithms that combine several base models to produce a better predictive model. To decrease variance, a bagging ensemble method can be used and to decrease bias a boosting ensemble method can be used. A boosting method converts weak learners to strong ones^50–52^. Usually, decision stumps are used as the base weak learners, but this is not always the case. Most Boosting methods build models in a stage-wise fashion and they generalize the model by optimizing an arbitrary differentiable loss function. Boosting methods also help prevent the problem of over-fitting to some extent. Additionally, Boosting methods solve the problems of a non-linear relation between target properties and features and help to deal with the collinearity among the features. Furthermore, most boosting methods provide the feature importance associated with the model. Feature importance is important to conclude which features influence Young’s modulus the most. Boosting methods are affected by the presence of outliers. Hence, it is recommended to perform outlier analysis before training the data.

### Feature importance

**Figure 2:**
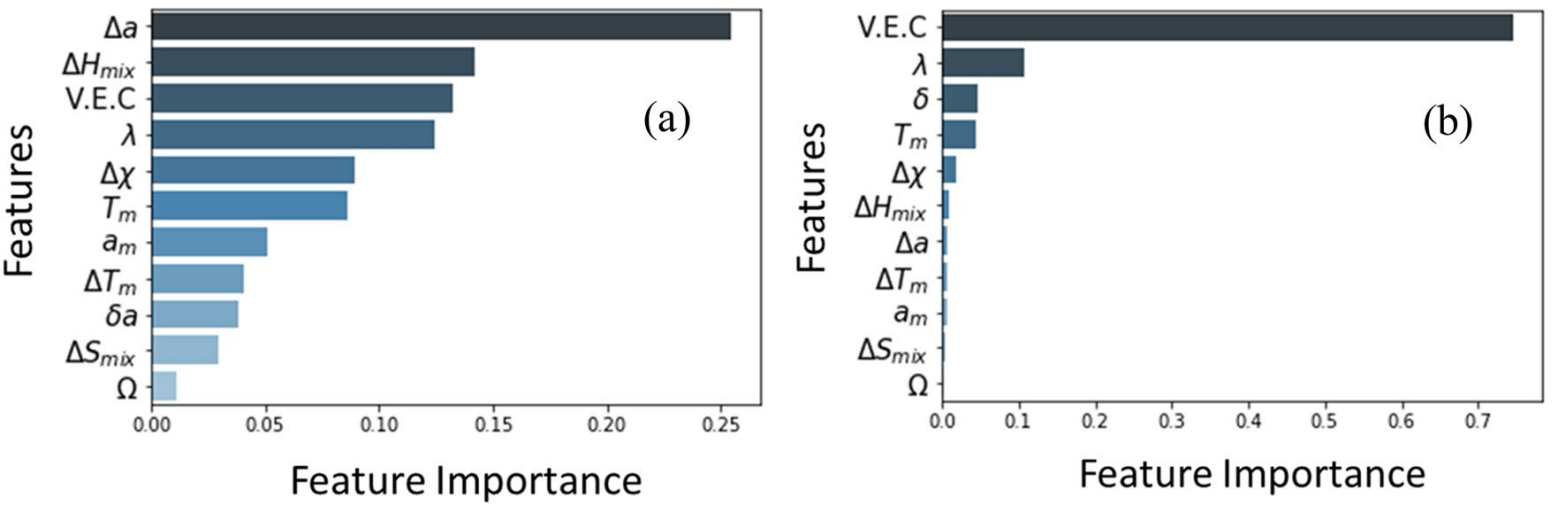
Feature importance for (a) larger training set containing both refractory and non-refractory alloys, (b) smaller training set containing only refractory alloys.

After training the models on both data sets containing refractory and non-refractory alloys using the optimized hyper-parameters, we determined the feature importance associated with the Gradient Boosting model. Feature importance is simply the score assigned to the features based on how useful they are at predicting a target variable. The feature importance for the larger data set containing both the refractory and non-refractory alloys, and smaller data set only with refractory alloys are presented in Fig. 2a,b, respectively. From feature importance, it is clear that the sequence of the features is not identical for both data sets. However, the smaller training data set showed better prediction accuracy as indicated in Table 2. Hence, we selected the important features generated from the smaller data set presented in Fig. 2b. In the next paragraph, we are going to explain the physical significance of some of the important features for the Young’s modulus of CCAs.

We found that VEC was the most important feature and had importance higher than 0.7. While it is not shown here, it is important to mention that other ML models i.e. XGBoost and RF showed good prediction capabilities and identified the VEC as the most important feature with an importance of more than 0.7. In the elastic limit and at a constant value of Poisson’s ratio*,* the Young’s modulus is related to the bulk modulus (Eq. 1) and hence we will explain the physics of the Young’s modulus dependence on VEC by exploring the physical relationship between bulk modulus and VEC^53,54^.

1 $$K = \frac{E}{3(1 - 2v)}$$

**Figure 3:**
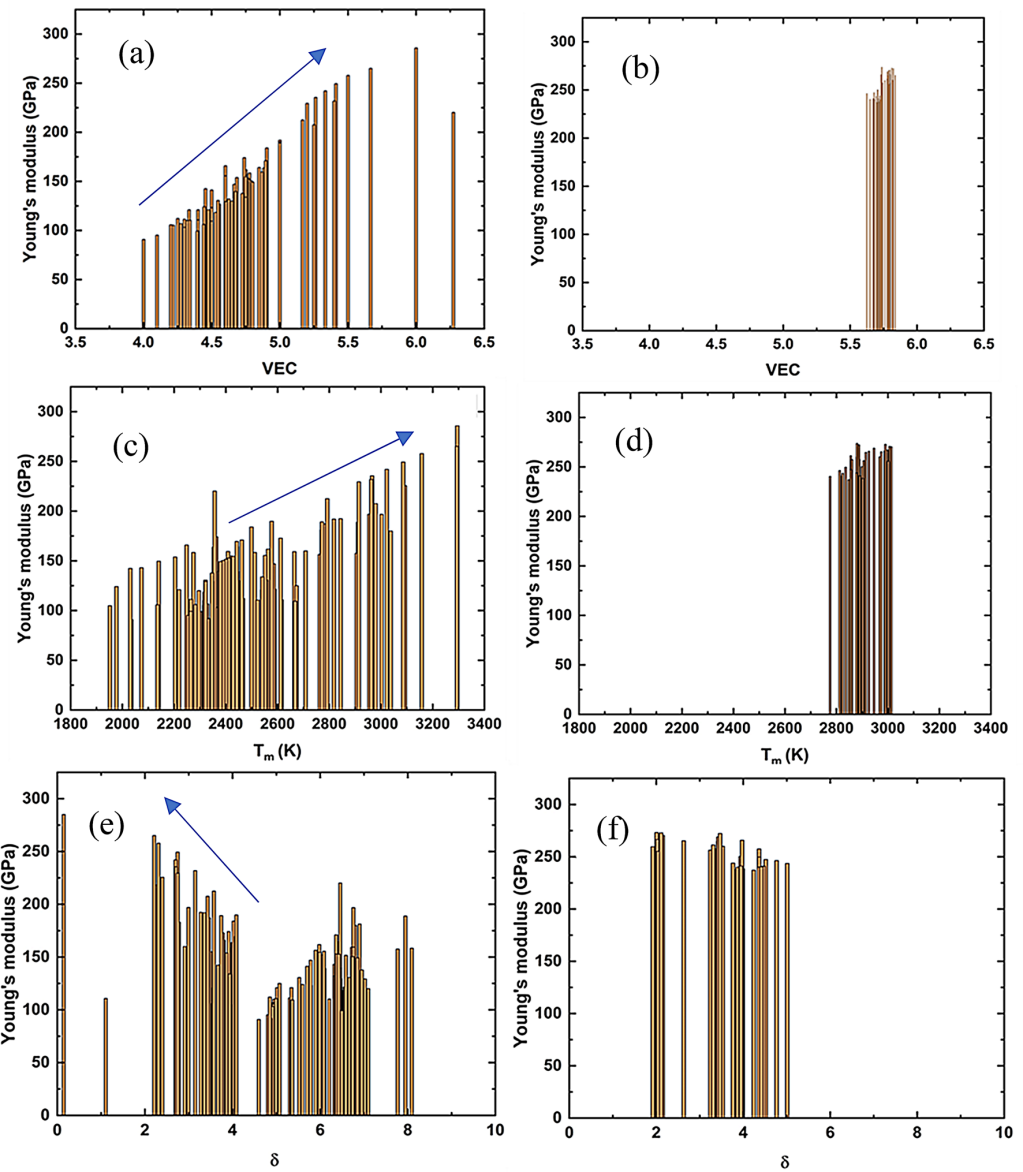
Impact of of some prominent features on Young’s modulus. (a) Relation between Young’s modulus and VEC for training set and (b) for experimental validation set. (c) Relation between melting temperature and Young’s modulus for training set and (d) for experimental validation set. (e) Relation between the difference of atomic radii and Young’s modulus for training set and (f) for experimental validation set.

Here, *K*, *E* and $v$ are the bulk modulus, Young’s modulus and Poisson’s ratio, respectively. Gilman et al.^53,54^, reported that materials with higher valence electron density (VED) (valence electrons/unit volume) possess higher bulk modulus. As the number of valence electrons increases, the bulk modulus increases, and it decreases as the atomic size increases. The bulk modulus is determined predominantly by the resistance of the valence electrons to compression. In a metallic system, electrons behave like a dense gas, or liquid, with only a very small amount of viscosity. Hence, the greater the electron density, the more the resistance to compression, and the higher the bulk modulus and the Young’s modulus. For instance, osmium, possesses a VED 17% higher than for diamond and correspondingly exhibits a bulk modulus 4% greater as well^53,54^. Though we considered VEC instead of VED in this work, it still follows the upward trend of Young’s modulus both for training and validation data sets with VEC as presented in Fig. 3a,b. Our calculated feature importance indicates that the melting point of alloys, which is an indirect metric of bond strength^34,55^, has an impact on Young’s modulus, which generally increases with increasing melting temperature as presented in Fig. 3c,d. The geometrical parameter λ, which is a function of mixing entropy ($\Delta S_{mix}$) and the difference in atomic radii (δ) has a significant impact on Young’s modulus. The δ parameter has an impact on cohesive energy and Young’s modulus increases with increasing cohesive energy^56,57^. In our case, we have seen that a lower value of δ results in higher Young’s modulus as presented in Fig. 3e,f. The difference in atomic radius influences the distribution of alloying elements and metallic bond energy. The electronegativity has an impact on the electron density of atoms and the larger value of electronegativity result in a higher Young’s modulus of metallic alloys^58^. Additionally, larger electronegativity differences ($\Delta \chi$) and higher mixing enthalpy ($\Delta H_{mix}$) increases the probability of formation of intermetallic brittle phases, which have lower Young’s modulus. Therefore, these two parameters could play an important role to determine Young’s modulus of CCAs^34^.

It is important to mention that Roy et al.^34^ predicted Young’s modulus of low, medium and high entropy alloys composed of 5 elements by employing Gradient Boosting method and found that average melting temperature (*T*_*m*_) was the most important feature without considering the impact of VEC. Corresponding MAE for their study was 23.59 GPa. In this study, we achieved significantly better performance (MAE = 6.15 GPa) by considering VEC in the feature sets. From the above discussion, we propose that VEC is the most important feature that determines the Young’s modulus of this refractory alloy system. Therefore, it is essential to include VEC as a key parameter in the design of new CCAs with tailored Young’s modulus.

### Experimental validation

**Figure 4:**
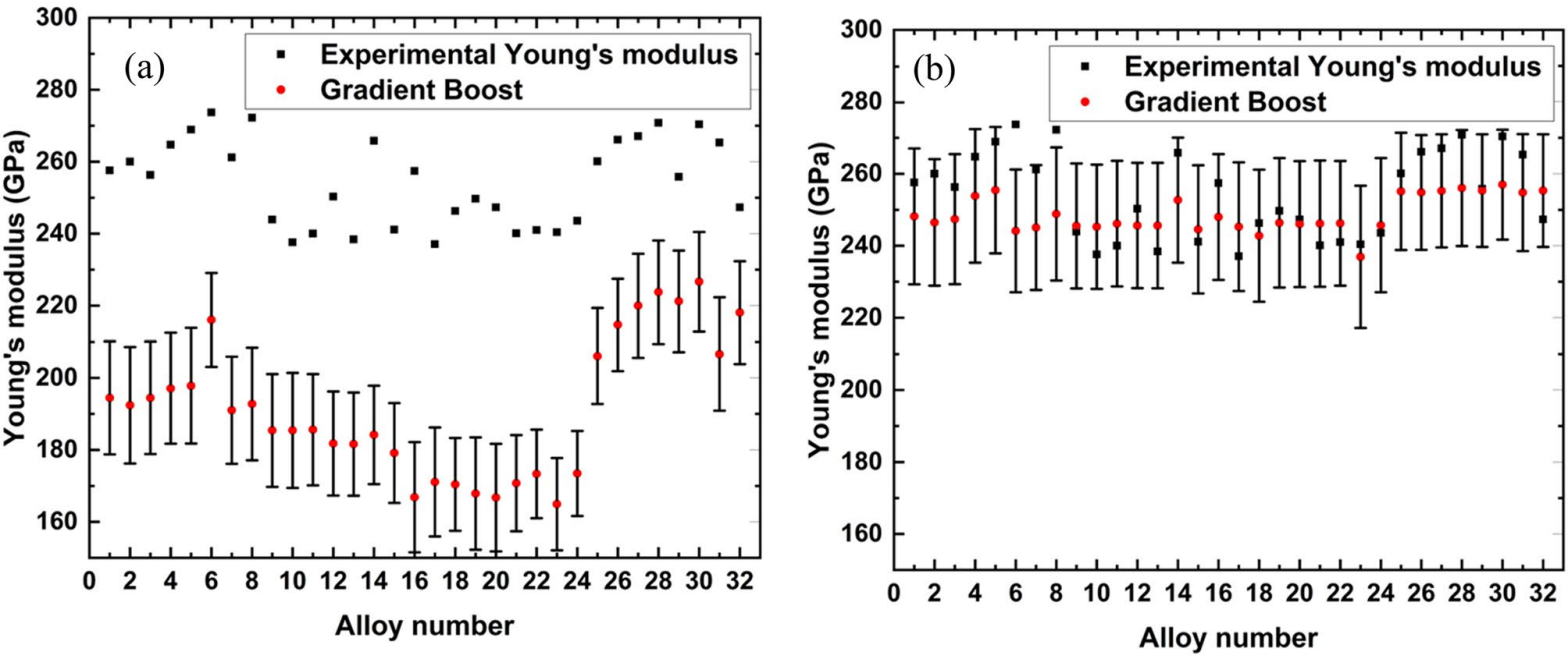
Young's Modulus Prediction by Gradient Boosting model when trained (a) with data containing both refractory and non-refractory alloys and (b) with only refractory alloys.

We finally used the trained Gradient Boosting model to predict Young's modulus of unseen CCAs, which are the experimentally synthesized 32 CCAs mostly composed of Mo–Ta–Ti–W–Zr elements. As the experimental validation alloys are all refractory alloys, we examined how the types of training sets have impact on the prediction of Young’s modulus. When we trained the Gradient Boosting model with larger data set containing both refractory and non-refractory alloys the predictions of the Young’s modulus were significantly off compared to experimentally measured Young’s modulus as presented in Fig. 4a. The predicted value consistently underestimated the experimental value. In contrast, we have achieved excellent predictions when we consider only the refractory alloys to train the Gradient Boosting model as presented in Fig. 4b. Only 2 predictions (alloy numbers 6 and 8) out of 32 alloys are outside of 68.3% confidence interval (± σ, where σ is the standard deviation of each prediction. Table 3 presents the actual value of experimental Young’s modulus, mean prediction of Young’s modulus with the percentage of error and standard deviation when the model was trained with refractory alloys. 26 of the alloys had errors ≤ 5% and a few of the predictions are almost identical compared to experimental values.

**Table 3:** Predicted Young's modulus with percentage of error and standard deviation from Gradient Boosting model trained with data containing refractory alloys.

Alloy number	Alloy composition (actual at. % compositions by EDS)	Experimental Young's modulus (GPa)	Mean prediction	% Error	Standard deviation (± σ)
1	Mo_85.25_Ta_9.52_Ti_2.29_Zr_2.94_	257.6	248.2	3.7	18.9
2	Mo_82.23_W_1.29_Ta_9.46_Ti_3.27_Zr_3.36_Al_0.39_	260	246.5	5.2	17.6
3	Mo_82.93_W_2_Ta_9.89_Ti_2.4_Zr_2.72_Al_0.05_	256.3	247.4	3.5	18.1
4	Mo_80.67_W_3.3_Ta_10.34_Ti_2.45_Zr_3.13_Al_0.05_Cr_0.06_	264.7	253.9	4.1	18.6
5	Mo_76.41_W_7.23_Ta_10.69_Ti_2.33_Zr_3.17_Al_0.16_	268.9	255.5	5.0	17.6
6	Mo_78.92_W_4.27_Ta_10.72_Ti_2.7_Al_3.39_	273.7	244.1	10.8	17.1
7	Mo_84.31_W_2.48_Ta_5.84_Ti_2.64_Zr_2.95_Al_1.79_	261.2	245.1	6.2	17.3
8	Mo_85.25_W_3.05_Ta_5.51_Ti_2.28_Zr_3.39_Al_0.23_Cr_0.29_	272.2	248.8	8.6	18.5
9	Mo_79.73_W_0.09_Ta_12.36_Ti_3.92_Zr_3.88_Cr_0.03_	243.9	245.5	− 0.7	17.4
10	Mo_78.53_W_1.06_Ta_12.53_Ti_3.68_Zr_4.18_Cr_0.03_	237.6	245.3	− 3.2	17.3
11	Mo_78.58_W_2.14_Ta_11.19_Ti_3.79_Zr_4.3_	240	246.1	− 2.6	17.5
12	Mo_75.86_W_3.13_Ta_12.65_Ti_3.89_Zr_4.47_	250.3	245.6	1.9	17.4
13	Mo_75.66_W_3.69_Ta_12.2_Ti_3.8_Zr_4.65_	238.4	245.6	− 3.0	17.4
14	Mo_73.77_W_7.67_Ta_10.17_Ti_3.7_Zr_4.69_	265.8	252.7	4.9	17.4
15	Mo_81.5_W_1.63_Ta_6.37_Ti_3.9_Zr_4.51_Al_1.96_Cr_0.13_	241.1	244.6	− 1.4	17.8
16	Mo_78.86_W_2.93_Ta_7.48_Ti_3.69_Zr_5.36_Cr_1.68_	257.4	248.0	3.7	17.5
17	Mo_79.92_Ta_9.87_Ti_4.69_Zr_5.45_Cr_0.07_	237.1	245.3	− 3.5	17.9
18	Mo_76.31_W_0.41_Ta_9.3_Ti_6.22_Zr_7.29_Al_0.37_Cr_0.08_	246.3	242.8	1.4	18.4
19	Mo_80.87_W_1.02_Ta_6.98_Ti_5.23_Zr_5.88_Al_0.03_	249.7	246.4	1.3	18.0
20	Mo_76.47_W_3.17_Ta_8.64_Ti_5.25_Zr_6.45_Cr_0.02_	247.3	246.0	0.5	17.5
21	Mo_73.61_W_5.27_Ta_10.49_Ti_4.71_Zr_5.93_	240.1	246.2	− 2.5	17.5
22	Mo_71.98_W_6.62_Ta_9.97_Ti_5.06_Zr_6.32_Cr_0.06_	241	246.2	− 2.2	17.3
23	Mo_80.03_W_1.49_Ta_4.47_Ti_5.24_Zr_6.01_Al_2.73_Cr_0.04_	240.4	236.9	1.4	19.8
24	Mo_78.09_W_3.06_Ta_4.93_Ti_4.92_Zr_7.9_Cr_1.1_	243.6	245.7	− 0.9	18.7
25	Mo_81.65_W_0.17_Ta_18.12_Ti_0.05_	260.1	255.1	1.9	16.3
26	Mo_78.35_W_1.61_Ta_20.03_	266.1	254.8	4.2	15.9
27	Mo_76.96_W_2.93_Ta_20_Ti_0.1_	267.1	255.3	4.4	15.7
28	Mo_75.99_W_3.83_Ta_20.18_	270.8	256.1	5.4	16.1
29	Mo_76.32_W_3.14_Ta_20.48_Ti_0.05_Cr_0.01_	255.8	255.3	0.2	15.7
30	Mo_74.54_W_4.2_Ta_21.25_	270.4	257.0	5.0	15.3
31	Mo_80.97_W_3.88_Ta_14.61_Zr_0.04_Al_0.49_	265.3	254.8	4.0	16.3
32	Mo_77.21_W_4.17_Ta_17.69_Ti_0.34_Zr_0.07_Al_0.1_Cr_0.41_	272.8	255.3	6.4	15.7

From Fig. 4 and Table 3 we conclude that the quality of the training data is very important to predict the target property accurately. We have a larger training set (154 alloys) with refractory and non-refractory alloys. On the other hand, we have a smaller training set (96 alloys) only with refractory alloys. Since the training set was more homogeneous for the smaller data set, we achieved better predictions. Moreover, the predicted Young’s modulus followed the trend with the experimental Young’s modulus with some exceptions as presented in Fig. 4b. Therefore, it is not only the size of the training data but also the quality and relevance of the training data that are important for better predictions.

## Conclusion

We have presented an approach that uses ML with high throughput experimental synthesis and mechanical testing of alloys to predict the Young’s modulus of CCAs reliably. We conclude that among the eight ML models we used, Gradient Boosting had the best predictive strength. The prediction of Young’s modulus was influenced by the model chosen and by the composition of training data. Our experimental validation set was composed of refractory alloys, and when the models were trained with data containing only refractory alloys, the predictions were closer to the experimental values. This shows that when training ML models to predict characteristics of alloys, it is advantageous to include alloys of similar composition in the training data set. The valence electron concentration is the most important feature governing the Young’s modulus of refractory CCAs and can be used to rapidly screen alloys. Since feature importance also appears to be influenced by the choice of training data set, it is important to choose carefully the training data set based on the type of alloy being studied and validate against high-quality experimental data of known provenance. The integration of experimental synthesis and testing, machine learning, and physics-based interpretation demonstrated in this work holds considerable promise for alloy design and property prediction.

## Supplementary Information

Supplementary Tables.

## Data Availability

The data that support the findings of this study are available from the corresponding author upon reasonable request.

## Code availability

The codes that support the findings of this study are available from the corresponding author upon reasonable request.

## References

[1] Huang SC (2017). Mechanical properties of zirconium-based random alloys: Alloying elements and composition dependencies. Comput. Mater. Sci..

[2] Inoue A (2015). Marzouki, development and applications of highly functional Al-based materials by use of metastable phases. Mater. Res..

[3] Abdelaziz MH, Paradis M, Samuel AM, Doty HW, Samuel FH (2017). Effect of aluminum addition on the microstructure, tensile properties, and fractography of cast Mg-based alloys. Ann. Mater. Sci. Eng..

[4] Schinhammer M, Hänzi AC, Löffler JF, Uggowitzer PJ (2010). Design strategy for biodegradable Fe-based alloys for medical applications. Acta Biomater..

[5] Long H, Mao S, Liu Y, Zhang Z, Han X (2018). Microstructural and compositional design of Ni-based single crystalline superalloys—A review. J. Alloy. Compd..

[6] Hayama AOF (2014). Effects of composition and heat treatment on the mechanical behavior of Ti–Cu alloys. Mater. Des..

[7] Yeh JW (2004). Nanostructured highentropy alloys with multiple principal elements: Novel alloy design concepts and outcomes. Adv. Eng. Mater..

[8] Cantor B, Chang ITH, Knight P, Vincent AJB (2004). Microstructural development in equiatomic multicomponent alloys. Mater. Sci. Eng. A.

[9] Yim D, Kim HS (2017). Fabrication of the high-entropy alloys and recent research trends: A review. Korean J. Met. Mater..

[10] Ren B (2012). Corrosion behavior of CuCrFeNiMn high entropy alloy system in 1 M sulfuric acid solution. Mater. Corros..

[11] Kang YB, Shim SH, Lee KH, Hong SI (2018). Dislocation creep behavior of CoCrFeMnNi high entropy alloy at intermediate temperatures. Mater. Res. Lett..

[12] Fu ZQ, MacDonald BE, Monson TC (2018). Influence of heat treatment on microstructure, mechanical behavior, and soft magnetic properties in an fcc-based Fe29Co28Ni29Cu7Ti7 high-entropy alloy. J. Mater. Res..

[13] Tikhonovsky MA, Salishchev GA, Yurchenko NY, Stepanov ND, Zherebtsov SV (2018). Aging behavior of the HfNbTaTiZr high entropy alloy. Mater. Lett..

[14] Qiu Y (2017). A lightweight single-phase AlTiVCr compositionally complex alloy. Acta Mater..

[15] Jensen JK (2016). Characterization of the microstructure of the compositionally complex alloy Al1Mo0.5Nb1Ta0.5Ti1Zr1. Scr. Mater..

[16] Ye YF, Wang Q, Lu J, Liu CT, Yang Y (2016). High-entropy alloy: Challenges and prospects. Mater. Today.

[17] Miracle DB, Senkov ON (2017). A critical review of high entropy alloys and related concepts. Acta Mater..

[18] Ma D, Grabowski B, Körmann F, Neugebauer J, Raabe D (2015). Ab initio, thermodynamics of the CoCrFeMnNi high entropy alloy: Importance of entropy contributions beyond the configurational one. Acta Mater..

[19] Zhang C, Zhang F, Chen S, Cao W (2012). Computational thermodynamics aided high-entropy alloy design. J. Occup. Med..

[20] Jiang C, Uberuaga BP (2016). Efficient ab initio modeling of random multicomponent alloys. Phys. Rev. Lett..

[21] Saal JE, Berglund IS, Sebastian JT, Liaw PK, Olson GB (2017). Equilibrium high entropy alloy phase stability from experiments and thermodynamic modeling. Scr. Mater..

[22] Lederer Y, Toher C, Vecchio KS, Curtarolo S (2018). The search for high entropy alloys: A high-throughput ab-initio approach. Acta Mater..

[23] Sanchez JM, Vicario I, Albizuri J, Guraya T, Garcia JC (2018). Phase prediction, microstructure and highhardness of novel light-weight high entropy alloys. J. Mater. Res. Technol..

[24] Tapia AJSF, Yim D, Kim HS, Lee BJ (2018). An approach for screening single phase high-entropy alloys using an inhouse thermodynamic database. Intermetallics.

[25] Senkov ON, Miller JD, Miracle DB, Woodward C (2015). Accelerated exploration of multiprincipal element alloys with solid solution phases. Nat. Commun..

[26] Bojarski, M. *et al*. End to end learning for self-driving cars. Preprint at arXiv:1604.07316 (2016).

[27] He K, Zhang X, Ren S, Sun J, Bajcsy R, Hager G (2015). Delving deep into rectifiers: Surpassing humanlevel performance on ImageNet classification. 2015 IEEE International Conference on Computer Vision (ICCV).

[28] Pazzani M, Billsus D (1997). Learning and revising user profiles: The identification of interesting web sites. Mach. Learn..

[29] Chan PK, Stolfo SJ, Agrawal R, Stolorz P, Piatetsky G (1998). Toward scalable learning with non-uniform class and cost distributions: A case study in credit card fraud detection. KDD’98 Proc. Fourth International Conference on Knowledge Discovery and Data Mining.

[30] Rickman JM, Balasubramanian G, Marvel CJ, Chan HM, Burton M-T (2020). Machine learning strategies for high-entropy alloys. J. Appl. Phys..

[31] Singh P, Sharma A, Smirnov AV, Diallo MS, Ray P, Balasubramanian G, Johnson DD (2018). Design of high-strength refractory complex solid-solution alloys. npj Comput. Mater..

[32] Singh P, Smirnov AV, Alam A, Johnson DD (2020). First-principles prediction of incipient order in arbitrary high-entropy alloys: Exemplified in Ti0.25CrFeNiAlx. Acta Mater..

[33] Singh P (2020). Vacancy-mediated complex phase selection in high entropy alloys. Acta Mater..

[34] Roy A, Babuska T, Krick B, Balasubramanian G (2020). Machine learned feature identification for predicting phase and Young’s modulus of low-, medium- and high-entropy alloys. Scr. Mater..

[35] Islam N, Huang W, Zhuang HL (2018). Machine learning for phase selection in multi-principal element alloys. Comput. Mater. Sci..

[36] Senkov O, Miracle D, Chaput K, Couzinie J (2018). Development and exploration of refractory high entropy alloys—A review. J. Mater. Res..

[37] Li W, Liu P, Liaw PK (2018). Microstructures and properties of high-entropy alloy films and coatings: A review. Mater. Res. Lett..

[38] Couzinié J-P, Senkov ON, Miracle DB, Dirras G (2018). Comprehensive data compilation on the mechanical properties of refractory high-entropy alloys. Data Brief..

[39] Fang S, Xiao X, Xia L, Li W, Dong Y (2003). Relationship between the widths of supercooled liquid regions and bond parameters of Mg-based bulk metallic glasses. J. Non-Cryst. Solids.

[40] Guo S, Ng C, Lu J, Liu CT (2011). Effect of valence electron concentration on stability of fcc or bcc phase in high entropy alloys. J. Appl. Phys..

[41] Takeuchi A, Inoue A (2005). Classification of bulk metallic glasses by atomic size difference, heat of mixing and period of constituent elements and its application to characterization of the main alloying element. Mater. Trans..

[42] Yang X, Zhang Y (2012). Prediction of high-entropy stabilized solid-solution in multi-component alloys. Mater. Chem. Phys..

[43] Singh AK, Kumar N, Dwivedi A, Subramaniam A (2014). A geometrical parameter for the formation of disordered solid solutions in multi-component alloys. Intermetallics.

[44] Senkov ON, Wilks GB, Miracle DB, Chuang CP, Liaw PK (2010). Refractory high-entropy alloys. Intermetallics.

[45] Breiman, L. Arcing The Edge. Technical Report 486. Statistics Department, University of California, Berkeley (1997).

[46] Pedregosa F (2011). Scikit-learn: Machine learning in Python. J. Mach. Learn. Res..

[47] Tianqi, C. & Carlos, G. XGBoost: A scalable tree boosting system. In *Proceedings of the 22nd ACM SIGKDD International Conference on Knowledge Discovery and Data Mining*, 785–794 (2016).

[48] Mamun O, Wenzlick M, Hawk J (2021). A machine learning aided interpretable model for rupture strength prediction in Fe-based martensitic and austenitic alloys. Sci. Rep..

[49] Mamun O, Wenzlick M, Sathanur A (2021). Machine learning augmented predictive and generative model for rupture life in ferritic and austenitic steels. npj Mater. Degrad..

[50] Schapire RE (1990). The strength of weak learnability. Mach. Learn..

[51] Friedman JH (2001). Greedy function approximation: A gradient boosting machine (PDF). Ann. Stat..

[52] Freund Y, Schapire RE (1997). A decision-theoretic generalization of on-line learning and an application to boosting. J. Comput. Syst. Sci..

[53] Gilman JJ (2003). Electronic Basis of the Strength of Materials, Chapter 12.

[54] Gilman JJ, Cumberland RW, Kaner RB (2006). Design of hard crystals. Int. J. Refract. Met. Hard Mater..

[55] Rickman JM (2018). Data analytics and parallel-coordinate materials property charts. npj Comput. Mater..

[56] Roy A, Sreeramagiri P, Babuska T, Krick B, Ray PK, Balasubramanian G (2021). Lattice distortion as an estimator of solid solution strengthening in high-entropy alloys. Mater. Charact..

[57] Pettifor DG, Cahn RW, Haasen P (1983). Electron theory of metals. Physical Metallurgy.

[58] Li K, Kang C, Xue D (2012). Electronegativity calculation of bulk modulus and band gap of ternary ZnO-based alloys. Mater. Res. Bull..

