# Supplementary material for: Machine learning assisted prediction of the Young’s modulus of compositionally complex alloys
Source: Sci Rep. 2021 Aug 25;11:17149. doi: 10.1038/s41598-021-96507-0 (PMC8387451; doi:10.1038/s41598-021-96507-0)
Supplement: Supplementary file 1 — Supplementary Tables. [file 41598_2021_96507_MOESM1_ESM.docx]

**Machine Learning Assisted Prediction of the Young’s Modulus of Compositionally Complex Alloys**

Hrishabh Khakurel^a, 1^, M. F. N. Taufique^b, 1*^, Ankit Roy^c^, Ganesh Balasubramanian^d^, Gaoyuan Ouyang^e^, Duane D. Johnson^e^_,_ Ram Devanathan^b^

^a^ Department of Mathematics, The University of Texas at Arlington, Arlington, TX 76019 , USA

^b^ Pacific Northwest National Laboratory, Richland, WA 99354, USA

^c^ Department of Mechanical Engineering and Mechanics, Lehigh University, Bethlehem, PA18015, USA

^d^ Ames Laboratory, United States Department of Energy, Ames, IA 50011, USA

Table 1: Training data set for refractory and non-refractory alloys.

| Alloy No | Alloy | Young's Modulus (GPa) |
| --- | --- | --- |
| 1 | Al0.5CoCrCuFeNi | 161.1 |
| 2 | Al0.3CoCrFeNi | 168 |
| 3 | Al0.5CrCuFeNi2 | 172.1 |
| 4 | CoCrFeNi | 173.9 |
| 5 | NiCoCr | 174.4 |
| 6 | FeNiCo | 173.8 |
| 7 | AlCoCrCuFeNi | 171.7 |
| 8 | Al0.5CoCrCuFeNi | 173.3 |
| 9 | AlCo0.5CrCuFeNi | 71.6 |
| 10 | AlCoCr0.5CuFeNi | 161.1 |
| 11 | AlCoCrCu0.5FeNi | 121.1 |
| 12 | AlCoCrCuFe0.5Ni | 178.6 |
| 13 | AlCoCrCuFeNi0.5 | 115.5 |
| 14 | CuNiCoFeCrAl0.5 | 174.4 |
| 15 | CuNiCoFeCrAl0.5V0.2 | 141.1 |
| 16 | CuNiCoFeCrAl0.5V0.4 | 71.9 |
| 17 | CuNiCoFeCrAl0.5V0.6 | 130.9 |
| 18 | CuNiCoFeCrAl0.5V0.8 | 166.7 |
| 19 | CuNiCoFeCrAl0.5V1.0 | 127.8 |
| 20 | CuNiCoFeCrAl0.5V1.2 | 257.3 |
| 21 | CuNiCoFeCrAl0.5V1.4 | 218 |
| 22 | CuNiCoFeCrAl0.5V1.6 | 130.5 |
| 23 | CuNiCoFeCrAl0.5V1.8 | 136.6 |
| 24 | CuNiCoFeCrAl0.5V2.0 | 128 |
| 25 | FeNiCrCuCo | 112 |
| 26 | FeNiCrCuMo | 81.9 |
| 27 | FeNiCrCoAl1.5 | 121.1 |
| 28 | Fe NiCrCoAl2 | 141.7 |
| 29 | FeNiCrCoAl2.5 | 141.6 |
| 30 | FeNiCrCoAl3 | 141.7 |
| 31 | FeNiCrCuZr | 141.5 |
| 32 | CuNiCoFeMn | 141.1 |
| 33 | CuNiCoFeMnSn0.03 | 141.4 |
| 34 | CuNiCoFeMnSn0.05 | 141.6 |
| 35 | CuNiCoFeMnSn0.08 | 137.2 |
| 36 | CuNiCoFeMnSn0.1 | 137.6 |
| 37 | CuNiCoFeMnSn0.2 | 139.5 |
| 38 | CuNi2FeCrAl0.2 | 137.9 |
| 39 | CuNi2FeCrAl0.3 | 202 |
| 40 | CuNi2FeCrAl0.4 | 178.6 |
| 41 | CuNi2FeCrAl0.5 | 78.1 |
| 42 | CuNi2FeCrAl0.8 | 139.15 |
| 43 | Al1.25CoCrFeNi | 55.6 |
| 44 | Al1.5CoCrFeNi | 98.6 |
| 45 | Al2CoCrFeNi | 128.3 |
| 46 | Al0.5CoCrCuFeNiTi0.2 | 76.5 |
| 47 | AlCoCrFeNi2 | 148 |
| 48 | AlCoCr1.2FeNi2 | 150 |
| 49 | AlCoCr1.4FeNi3 | 157 |
| 50 | AlCoCr1.6FeNi4 | 88.46 |
| 51 | AlCoCr1.8FeNi5 | 105 |
| 52 | AlCoCr2.0FeNi6 | 162 |
| 53 | AlCoCr2.2FeNi7 | 102 |
| 54 | Al0.5CrFeMnNi | 117 |
| 55 | Al0.6CrFeMnNi | 136 |
| 56 | Al0.7CrFeMnNi | 174 |
| 57 | Al0.8CrFeMnNi | 232.744 |
| 58 | FeMnNi | 182 |
| 59 | CoMnNi | 190 |
| 60 | CoFeNi | 161 |
| 61 | CoCrNi | 226 |
| 62 | Co4Ni4Cr | 223 |
| 63 | Co19Ni19Cr1 | 219 |
| 64 | CoFeMnNi | 186 |
| 65 | CoCrFeNi | 210 |
| 66 | CoCr4Ni4 | 240 |
| 67 | CoCrFeMnNi | 202 |
| 68 | CoCrFeNiV | 233.7 |
| 69 | CoCrFeNiVAl0.5 | 209 |
| 70 | MoWZr | 157.5717 |
| 71 | MoTiTa | 159.9126 |
| 72 | WTaZr | 179.9365 |
| 73 | TiTaZr | 120.8907 |
| 74 | MoTiZr | 130.5883 |
| 75 | MoWTa | 265.0655 |
| 76 | MoWTi | 192.246 |
| 77 | WTiTa | 182.8289 |
| 78 | WTiZr | 121.5248 |
| 79 | MoTaZr | 181.26109 |
| 80 | MoWTaZr | 196.7513 |
| 81 | MoWTiZr | 159.15988 |
| 82 | MoWTiTa | 196.83719 |
| 83 | MoTaTiZr | 161.75 |
| 84 | WTiTaZr | 156.39548 |
| 85 | MoTaTiWZr | 152.04969 |
| 86 | Al0.25MoNbTiV | 163.6 |
| 87 | Al0.25NbTaTiV | 130 |
| 88 | Al0.2MoTaTiV | 184 |
| 89 | Al0.3HfNbTaTiZr | 108.3 |
| 90 | Al0.3NbTa0.8Ti1.4V0.2Zr1.3 | 110.2 |
| 91 | Al0.4Hf0.6NbTaTiZr | 110 |
| 92 | Al0.5CrNbTi2V0.5 | 143 |
| 93 | Al0.5HfNbTaTiZr | 106.9 |
| 94 | Al0.5MoNbTiV | 158.4 |
| 95 | Al0.5NbTa0.8Ti1.5V0.2Zr | 111.3 |
| 96 | Al0.5NbTaTiV | 126.7 |
| 97 | Al0.6MoTaTiV | 174.1 |
| 98 | Al0.75HfNbTaTiZr | 105.3 |
| 99 | Al0.75MoNbTiV | 153.8 |
| 100 | Al1.5MoNbTiV | 142.4 |
| 101 | AlCr0.5NbTiV | 124.1 |
| 102 | AlMoNbTiV | 149.6 |
| 103 | AlMoTaTiV | 165.8 |
| 104 | AlNb1.5Ta0.5Ti1.5Zr0.5 | 105.7 |
| 105 | AlNbTaTiV | 121 |
| 106 | AlNbTiV | 104.8 |
| 107 | CoCrMoNbTi0.4 | 220.1 |
| 108 | Hf0.4Nb1.54Ta1.54Ti0.89Zr0.64 | 125 |
| 109 | Hf0.5Mo0.5NbTiZr | 123.1 |
| 110 | Hf0.5Nb0.5Ta0.5Ti1.5Zr | 106.6 |
| 111 | Hf0.75NbTa0.5Ti1.5Zr1.25 | 103.1 |
| 112 | HfMo0.25NbTaTiZr | 121 |
| 113 | HfMo0.5NbTaTiZr | 130.5 |
| 114 | HfMo0.5NbTiV0.5 | 131.9 |
| 115 | HfMoNbTaTiZr | 147 |
| 116 | HfMoNbTiZr | 139.2 |
| 117 | HfMoTaTiZr | 155.4 |
| 118 | HfNb0.18Ta0.18Ti1.27Zr | 95.2 |
| 119 | HfNbTaTiZr | 110.6 |
| 120 | HfNbTaZr | 109.3 |
| 121 | HfNbTiVZr | 99 |
| 122 | HfNbTiZr | 91.8 |
| 123 | HfTaTiZr | 112 |
| 124 | Mo0.1NbTiV0.3Zr | 106 |
| 125 | Mo0.3NbTiV0.3Zr | 118.4 |
| 126 | Mo0.3NbTiVZr | 119.9 |
| 127 | Mo0.5NbTiV0.3Zr | 129.4 |
| 128 | Mo0.5NbTiVZr | 129.2 |
| 129 | Mo0.7NbTiV0.3Zr | 139.4 |
| 130 | Mo0.7NbTiVZr | 137.7 |
| 131 | Mo1.3NbTiV0.3Zr | 164.2 |
| 132 | Mo1.3NbTiVZr | 159.4 |
| 133 | Mo1.5NbTiV0.3Zr | 171 |
| 134 | MoNbTaTi0.25W | 249.4 |
| 135 | MoNbTaTi0.5W | 242 |
| 136 | MoNbTaTi0.75W | 235.4 |
| 137 | MoNbTaTiV | 172.8 |
| 138 | MoNbTaTiVW | 212.5 |
| 139 | MoNbTaTiW | 229.4 |
| 140 | MoNbTaV | 187 |
| 141 | MoNbTaVW | 231.8 |
| 142 | MoNbTaW | 257.8 |
| 143 | MoNbTiV | 169.5 |
| 144 | MoNbTiV0.25Zr | 152.9 |
| 145 | MoNbTiV0.3Zr | 152.7 |
| 146 | MoNbTiV0.5Zr | 151.6 |
| 147 | MoNbTiV0.75Zr | 150.3 |
| 148 | MoNbTiVZr | 149.2 |
| 149 | MoNbTiZr | 154.5 |
| 150 | MoTaTiV | 189.8 |
| 151 | NbTaTiV | 133.8 |
| 152 | NbTaTiVW | 189.2 |
| 153 | NbTaVW | 207.5 |
| 154 | NbTiV0.3Zr | 99.2 |

Table 2: Training data only with refractory alloys.

| Alloy No | Alloy | Young's Modulus (GPa) |
| --- | --- | --- |
| 1 | TaNbHfZrTi | 49.88 |
| 2 | TiTa | 110.7408 |
| 3 | MoZr | 158.3784 |
| 4 | MoW | 285.8335 |
| 5 | WTa | 218.0652 |
| 6 | WZr | 188.7576 |
| 7 | MoTi | 154.9023 |
| 8 | TaZr | 141.1359 |
| 9 | TiZr | 90.7825 |
| 10 | WTi | 191.9181 |
| 11 | MoTa | 225.55 |
| 12 | MoWZr | 157.5717 |
| 13 | MoTiTa | 159.9126 |
| 14 | WTaZr | 179.9365 |
| 15 | TiTaZr | 120.8907 |
| 16 | MoTiZr | 130.5883 |
| 17 | MoWTa | 265.0655 |
| 18 | MoWTi | 192.246 |
| 19 | WTiTa | 182.8289 |
| 20 | WTiZr | 121.5248 |
| 21 | MoTaZr | 181.26109 |
| 22 | MoWTaZr | 196.7513 |
| 23 | MoWTiZr | 159.15988 |
| 24 | MoWTiTa | 196.83719 |
| 25 | MoTaTiZr | 161.75 |
| 26 | WTiTaZr | 156.39548 |
| 27 | MoTaTiWZr | 152.04969 |
| 28 | Al0.25MoNbTiV | 163.6 |
| 29 | Al0.25NbTaTiV | 130 |
| 30 | Al0.2MoTaTiV | 184 |
| 31 | Al0.3HfNbTaTiZr | 108.3 |
| 32 | Al0.3NbTa0.8Ti1.4V0.2Zr1.3 | 110.2 |
| 33 | Al0.4Hf0.6NbTaTiZr | 110 |
| 34 | Al0.5CrNbTi2V0.5 | 143 |
| 35 | Al0.5HfNbTaTiZr | 106.9 |
| 36 | Al0.5MoNbTiV | 158.4 |
| 37 | Al0.5NbTa0.8Ti1.5V0.2Zr | 111.3 |
| 38 | Al0.5NbTaTiV | 126.7 |
| 39 | Al0.6MoTaTiV | 174.1 |
| 40 | Al0.75HfNbTaTiZr | 105.3 |
| 41 | Al0.75MoNbTiV | 153.8 |
| 42 | Al1.5MoNbTiV | 142.4 |
| 43 | AlCr0.5NbTiV | 124.1 |
| 44 | AlMoNbTiV | 149.6 |
| 45 | AlMoTaTiV | 165.8 |
| 46 | AlNb1.5Ta0.5Ti1.5Zr0.5 | 105.7 |
| 47 | AlNbTaTiV | 121 |
| 48 | AlNbTiV | 104.8 |
| 49 | CoCrMoNbTi0.4 | 220.1 |
| 50 | Hf0.4Nb1.54Ta1.54Ti0.89Zr0.64 | 125 |
| 51 | Hf0.5Mo0.5NbTiZr | 123.1 |
| 52 | Hf0.5Nb0.5Ta0.5Ti1.5Zr | 106.6 |
| 53 | Hf0.75NbTa0.5Ti1.5Zr1.25 | 103.1 |
| 54 | HfMo0.25NbTaTiZr | 121 |
| 55 | HfMo0.5NbTaTiZr | 130.5 |
| 56 | HfMo0.5NbTiV0.5 | 131.9 |
| 57 | HfMoNbTaTiZr | 147 |
| 58 | HfMoNbTiZr | 139.2 |
| 59 | HfMoTaTiZr | 155.4 |
| 60 | HfNb0.18Ta0.18Ti1.27Zr | 95.2 |
| 61 | HfNbTaTiZr | 110.6 |
| 62 | HfNbTaZr | 109.3 |
| 63 | HfNbTiVZr | 99 |
| 64 | HfNbTiZr | 91.8 |
| 65 | HfTaTiZr | 112 |
| 66 | Mo0.1NbTiV0.3Zr | 106 |
| 67 | Mo0.3NbTiV0.3Zr | 118.4 |
| 68 | Mo0.3NbTiVZr | 119.9 |
| 69 | Mo0.5NbTiV0.3Zr | 129.4 |
| 70 | Mo0.5NbTiVZr | 129.2 |
| 71 | Mo0.7NbTiV0.3Zr | 139.4 |
| 72 | Mo0.7NbTiVZr | 137.7 |
| 73 | Mo1.3NbTiV0.3Zr | 164.2 |
| 74 | Mo1.3NbTiVZr | 159.4 |
| 75 | Mo1.5NbTiV0.3Zr | 171 |
| 76 | MoNbTaTi0.25W | 249.4 |
| 77 | MoNbTaTi0.5W | 242 |
| 78 | MoNbTaTi0.75W | 235.4 |
| 79 | MoNbTaTiV | 172.8 |
| 80 | MoNbTaTiVW | 212.5 |
| 81 | MoNbTaTiW | 229.4 |
| 82 | MoNbTaV | 187 |
| 83 | MoNbTaVW | 231.8 |
| 84 | MoNbTaW | 257.8 |
| 85 | MoNbTiV | 169.5 |
| 86 | MoNbTiV0.25Zr | 152.9 |
| 87 | MoNbTiV0.3Zr | 152.7 |
| 88 | MoNbTiV0.5Zr | 151.6 |
| 89 | MoNbTiV0.75Zr | 150.3 |
| 90 | MoNbTiVZr | 149.2 |
| 91 | MoNbTiZr | 154.5 |
| 92 | MoTaTiV | 189.8 |
| 93 | NbTaTiV | 133.8 |
| 94 | NbTaTiVW | 189.2 |
| 95 | NbTaVW | 207.5 |
| 96 | NbTiV0.3Zr | 99.2 |

Table 3: Optimized hyperparameters of different ML models.

| Model | Optimized Hyperparameters | |
| --- | --- | --- |
|  | Refractory and non-refractory dataset | Refractory dataset |
| Gradient Boost | learning_rate = 0.3  max_depth = 3  min_samples_leaf = 1 min_samples_split = 2  n_estimators =100 | learning_rate = 0.36  max_depth = 3  min_samples_leaf = 3  min_samples_split = 2  n_estimators = 100 |
| XGBoost | learning_rate = 0.3  max_delta_step = 0  max_depth = 4  min_child_weight = 4  n_estimators = 200 | learning_rate = 0.15  max_delta_step = 0  max_depth= 3  min_child_weight = 5  n_estimators = 100 |
| RF | max_depth =8  min_samples_leaf = 1  min_samples_split = 2  n_estimators = 700 | max_depth = 4  min_samples_leaf= 3  min_samples_split =2  n_estimators = 300 |
| Ada Boost | learning_rate = 0.3  n_estimators = 400 | learning_rate = 0.42  n_estimators = 500 |
| SVM | C = 290  gamma = 0.01 | C = 230  gamma = 0.060 |
| Lasso regression | alpha = 0.1 | alpha = 1.73 |
| Ridge regression | alpha = 0.1 | alpha = 63.30 |
| Gaussian process | alpha = 10 ×10^-11^ | alpha = 10 ×10^-13^ |
